# Current Issues in Molecular Biology Keep Evolving and Getting Better

**DOI:** 10.3390/cimb45100494

**Published:** 2023-09-26

**Authors:** Madhav Bhatia

**Affiliations:** Department of Pathology and Biomedical Science, University of Otago, Christchurch 8140, New Zealand; madhav.bhatia@otago.ac.nz; Tel.: +64-3-3786238; Fax: +64-3-3640009

Current Issues in Molecular Biology (CIMB) (https://www.mdpi.com/journal/cimb, accessed on 26 September 2023) has existed since 1999 and has evolved over the years. The journal is broad-based and publishes articles in diverse disciplines related to molecular biology, as evident from its scope (https://www.mdpi.com/journal/cimb/about, accessed on 26 September 2023) and various sections (https://www.mdpi.com/journal/cimb/sections, accessed on 26 September 2023). To keep the scope of the journal broad is a conscious decision, as we intend to publish articles from different disciplines and articles that are multi-disciplinary in nature. Recently, we have added ‘Molecular Pathology’ as one of the areas within the scope of the journal to reflect the goal of the journal, which is to publish high-quality papers in diverse disciplines related to molecular biology. The most important factor, from our perspective, is the quality of the work being published. In this regard, the journal continues to evolve and keeps getting better.

